# Meta-analytic connectivity modelling of functional magnetic resonance imaging studies in autism spectrum disorders

**DOI:** 10.1007/s11682-022-00754-2

**Published:** 2023-01-12

**Authors:** Alicia M. Goodwill, Li Tong Low, Peter T. Fox, P. Mickle Fox, Kenneth K. Poon, Sourav S. Bhowmick, S. H. Annabel Chen

**Affiliations:** 1 Physical Education and Sports Science Academic Group, National Institute of Education, Nanyang Technological University, Singapore, Singapore; 2 Centre for Research and Development in Learning, Nanyang Technological University, Singapore, Singapore; 3 Research Imaging Institute, University of Texas Health Science Center at San Antonio, San Antonio, TX USA; 4 Office of Education Research, National Institute of Education, Nanyang Technological University, Singapore, Singapore; 5 School of Computer Science and Engineering, Nanyang Technological University, Singapore, Singapore; 6 School of Social Sciences, Psychology, Nanyang Technological University, Singapore, Singapore; 7 Lee Kong Chian School of Medicine, Nanyang Technological University, Singapore, Singapore

**Keywords:** Autism spectrum disorder, Adult, fMRI, Meta-analysis, Meta-analytic connectivity modelling, BrainMap

## Abstract

Social and non-social deficits in autism spectrum disorders (ASD) persist into adulthood and may share common regions of aberrant neural activations. The current meta-analysis investigated activation differences between ASD and neurotypical controls irrespective of task type. Activation likelihood estimation meta-analyses were performed to examine consistent hypo-activated and/or hyper-activated regions for all tasks combined, and for social and non-social tasks separately; meta-analytic connectivity modelling and behavioral/paradigm analyses were performed to examine co-activated regions and associated behaviors. One hundred studies (mean age range = 18–41 years) were included. For all tasks combined, the ASD group showed significant (p < .05) hypo-activation in one cluster around the left amygdala (peak − 26, -2, -20, volume = 1336 mm^3^, maximum ALE = 0.0327), and this cluster co-activated with two other clusters around the right cerebellum (peak 42, -56, -22, volume = 2560mm^3^, maximum ALE = 0.049) Lobule VI/Crus I and the left fusiform gyrus (BA47) (peak − 42, -46, -18, volume = 1616 mm^3^, maximum ALE = 0.046) and left cerebellum (peak − 42, -58, -20, volume = 1616mm^3^, maximum ALE = 0.033) Lobule VI/Crus I. While the left amygdala was associated with negative emotion (fear) (z = 3.047), the left fusiform gyrus/cerebellum Lobule VI/Crus I cluster was associated with language semantics (z = 3.724) and action observation (z = 3.077). These findings highlight the left amygdala as a region consistently hypo-activated in ASD and suggest the potential involvement of fusiform gyrus and cerebellum in social cognition in ASD. Future research should further elucidate if and how amygdala-fusiform/cerebellar connectivity relates to social and non-social cognition in adults with ASD.

**Supplementary information:**

The online version contains supplementary material available at 10.1007/s11682-022-00754-2.

## Introduction

Autism spectrum disorders (ASD) are characterized by social-communicative symptoms and repetitive, restricted interests and behaviors (American Psychiatric Association, 2013). To date, much of the research and intervention programs have focused on the origins of ASD from a neurodevelopmental perspective. However, it is well established that impairments in many mental abilities can be lifelong, affecting approximately 1% of the adult population (Lange et al., 2015). With a scarcity of research to inform learning and behavioral interventions, adults with ASD are likely to experience persistent difficulties in obtaining and sustaining meaningful work and relationships (Scott et al., 2019), and are more susceptible to comorbid physical and mental health problems (Brondino et al., 2019; Eaves & Ho, 2008). This has prompted a call for more research to support the adult ASD population (Nicholas et al., 2017).

Over the past two decades, many functional magnetic resonance imaging (fMRI) studies have aimed to understand the aberrant brain-behavior relationships in various socio-emotional and neurocognitive processes in individuals with ASD. Morphological differences may undergo disturbed maturation throughout later childhood and adolescence (Nickl-Jockschat et al., 2012), which are often less pronounced in adults with ASD compared to with neurotypical controls (Courchesne et al., 2001). It is plausible that aberrant functional activations in the adult brain may manifest from this atypical maturation of brain morphology and contribute to cognitive and behavioural deficits throughout adulthood, with potential negative consequences on learning. For example, different functional activation patterns have been reported in widespread regions such as the cerebellum, striatum, limbic, prefrontal, and parieto-temporal regions across varying tasks of executive functioning, reward, and socio-emotional face processing (Gilbert et al., 2008; Janouschek et al., 2021; Kim et al., 2015; Murphy et al., 2017; Sato et al., 2019). However, there is little consensus on whether abberant regions of hypo- or hyper-activation converge across different task-paradigms, cognitive, and behavioural domains. Given much of the variation derived from fMRI studies can be related to the task stimuli employed and the cognitive processes in which these tasks probe, the putative brain bases for socio-emotional and neurocognitive impairments in adults with ASD remain unclear. Coordinate-based meta-analysis and meta-analytic connectivity modelling (MACM) can therefore serve as powerful tools to portray a more reliable picture of key regions of abberant hyper- and hypo-activation implicated in ASD.

To date, coordinate-based meta-analyses have supported the notion that disturbances in brain regions associated with aspects of social cognition (e.g., face processing) remain the most replicated finding in ASD (Di Martino et al., 2009; Dickstein et al., 2013; Philip et al., 2012; Nickl-Jockschat et al., 2015). More recently, meta-analyses looking at executive functions and reward processing in ASD have also noted aberrant activations within frontoparietal regions (May & Kana, 2020; Zhang et al., 2020) and the right striatum (Janouschek et al. 2021). Whilst these past meta-analyses have advanced our understanding of task-specific differences in brain activations in ASD, it remains unclear if certain regions are differentially activated independent of the task demands or stimuli presented. An understanding of differentially hypo- or hyper-activated brain regions regardless of task type would lead to more generalizability about consistent regions implicated in ASD which could be used as biomarkers and/or targets for intervention.

Moreover, it is now well established that aberrant functional connectivity across many regions, rather than any single region, contributes to the cognitive dysfunctions experienced by individuals with ASD (Vasa et al., 2016). This hypothesis is further supported by behavioral studies indicating a relationship between socio-emotional and non-social tasks (Haigh et al., 2018), which suggests the brain mechanisms underpinning these behaviors are not mutually exclusive and likely involve common networks. Therefore, the aim of this study was to provide an updated meta-analysis on regions consistently hypo- or hyper-activated in ASD (compared with neurotypical controls, [NC]), independent of the task stimuli used in the individual studies. Given the large number of studies published since previous reviews, we also performed an updated subgroup meta-analysis on social and non-social tasks separately. Our study also extends previous meta-analyses through the application of MACM based on a convergent seed region that is independent of the task-stimuli. Through this approach, we aim to provide novel insights into the functional connectivity and associated behavioral domains that may be implicated in ASD.

## Methods

### Literature search

A systematic search was conducted in PubMed/MEDLINE, PsycInfo, and the BrainMap database (initial search from inception to March 2020, last checked/updated for new papers March 2022) with combinations of the following free text search terms in title/abstract: (autism OR ASD OR Asperger OR “pervasive developmental disorder” OR PDD) AND (“functional magnetic resonance imaging” OR “functional MRI” OR fMRI OR imaging OR neuroimaging). Reference lists of existing ASD reviews on task-based fMRI were checked for additional studies. Inclusion criteria were: (1) original research studies (published journal articles) in English language; (2) mean age ≥ 18 years with a diagnosis of ASD (e.g., DSM-IV, ICD-10); (3) task-based fMRI activation; (4) direct between-group comparisons with a neurotypical control (NC) group; (5) whole-brain analyses; (6) coordinates reported in standard stereotactic space (MNI or Talairach); and (7) at least 7 participants in the smaller group. This was chosen based on recommendations from Tahmasian and Colleagues that samples smaller than this may yield invalid and/or non-replicable findings (Tahmasian et al., 2019). After initial title and abstract exclusion (LTL), two authors (LTL & AMG) independently screened full text for eligibility, and any disagreements were discussed and resolved.

### Data extraction

The following data were extracted from each study: sample size, age (mean and standard deviation), sex, and IQ for both ASD and NC groups, diagnosis, and diagnostic measure for the ASD group, fMRI task and stimuli, significant contrast(s), number of foci, reference space, correction for multiple comparisons, and source of coordinates. Studies were then further categorized by task type into social (e.g., theory of mind, face processing, emotional processing, language) and non-social (e.g., motor control, reward processing, executive functions, i.e., spatial attention, interference control, working memory). These categories were chosen to maintain adequate power for each meta-analysis (i.e., at least 17–20 experiments; Eickhoff et al., 2016) and were in line with previous meta-analyses on task-based fMRI studies that probed social and non-social behaviors (Di Martino et al., 2009; Dickstein et al., 2013). For the quantitative meta-analysis, the smaller sample size of the two groups (i.e., ASD or NC) and the stereotaxic coordinates of all significant contrasts for hypo-activated (i.e., ASD < NC) and hyper-activated (i.e., ASD > NC) regions were extracted and reported. If multiple significant contrasts were present within a study, or if the sample was used in more than one study, the coordinates were pooled and treated as one single experiment to adjust for multiple contrasts and reduce within-group bias (Müller et al., 2018; Turkeltaub et al., 2012). Prior to analyses, coordinates reported in Talairach space were converted into MNI space using the tal2icbm transform in the GingerALE ‘Convert Foci’ tool (Laird et al., 2010; Lancaster et al., 2007). One author (LTL) performed the initial data extraction and task categorization which was then checked by a second author (AMG). All included studies were coded into the BrainMap database and the coordinates for each experiment were additionally cross-checked with BrainMap to ensure data extraction and coding accuracy.

### Activation likelihood estimation (ALE)

Using GingerALE v3.0.2 software, ALE was performed on hypo-activated (between-group contrast ASD < NC) and hyper-activated (between-group contrast ASD > NC) results for: (1) all tasks combined, (2) socio-emotional tasks only, and (3) non-social tasks only. The ALE algorithm (Eickhoff et al., 2012, 2009; Turkeltaub et al., 2012) tests for spatial convergence of foci across studies, by taking the foci from each study and placing a 3D Gaussian probability distribution around each focus to estimate the likelihood of activation. This method accounts for the spatial uncertainty in neuroimaging results due to between-subject and between-template variability. The spatial probabilities of an activation being present at a given voxel are then combined to generate modelled activation (MA) maps for each study. The ALE value is computed at each voxel by taking the union of the MA maps across the studies. The resulting values are then applied on a histogram to be used as a null distribution. Thus, the ALE value indicates the spatial convergence across studies and is tested against the random convergence expected by chance (Eickhoff et al., 2012, 2009; Turkeltaub et al., 2012). Multiple comparisons were controlled for at the cluster level, which has been suggested as the most robust method to reduce type 1 error (Eickhoff et al., 2016; Müller et al., 2018). All ALE maps were thresholded at cluster-forming threshold *p* = .001 and cluster-level family-wise error (FWE) correction *p* = .05 after 1000 permutations (Müller et al., 2018). For all ALE meta-analyses, results are reported if two or more studies contributed to the significant cluster.

### Meta-analytic connectivity modelling (MACM)

Using Sleuth v3.0.4 and GingerALE v3.0.2 software, MACM was performed using significant cluster(s) from ALE as seed region(s). For each seed, BrainMap database was searched for all experiments on healthy subjects that reported at least one focus of activation within the seed region. Then, an ALE analysis was performed on the extracted experiments to test for spatial convergence across all reported foci. The statistical thresholds applied were the same as for ALE analyses (i.e., cluster-forming threshold *p* = .001; cluster-level FWE correction *p* = .05, 1000 permutations). The seed region would show high convergence, and convergence outside the seed region shows other brain regions with consistent co-activations with the seed region, which is reflective of task-based functional connectivity (Kotkowski et al., 2018; Laird et al., 2009; Robinson et al., 2012, 2010).

### Behavioral/paradigm analysis

Using Mango Behavioral Analysis v3.1 and Paradigm Analysis v1.6 plugins, we conducted functional decoding for the significant clusters from ALE and MACM. BrainMap consists of functional experiments characterized by 111 paradigm classes (to date), and the experiments are categorized into 5 behavioral domains (action, cognition, emotion, interception, and perception) and 60 subdomains (to date) (Fox & Lancaster, 2002; Fox et al., 2005; Laird et al., 2005; Vanasse et al., 2018). Behavioral/Paradigm Analysis uses BrainMap metadata to determine the behavioral domains/paradigm classes associated with a cluster. The probability of activation in a cluster given a domain/paradigm is tested against the baseline probability of the cluster activation. For each behavioral subdomain/paradigm class, z-scores are calculated and assessed for significance with a binomial test, and only z-scores ≥ 3.0 are considered significant (*p* < .05, Bonferroni corrected for multiple comparisons) (Lancaster et al., 2012).

### Assessment of robustness against noise studies

In order to address publication bias or the file draw problem in meta-analysis, it is important to determine bias towards significant findings being published over non-significant findings. In contrast to traditional meta-analysis which pools effect sizes of both significant and non-significant contrasts from published papers, fMRI meta-analysis only includes significant foci from each experiment. Therefore, non-significant experiments also need to be taken into consideration. Aiming to address the presence of this bias within the BrainMap database, Samartsidis and Colleagues (Samartsidis et al., 2020) used the BrainMap database and estimated that at least 6 per 100 studies or experiments would be missing due to prevalence of unpublished non-significant studies and non-significant experiments within published studies.

To address publication bias in this study, we used the adapted Fail-Safe N (FSN) (Acar et al., 2018), to assess the robustness of our ALE results against publication bias and individual study contribution. The adapted FSN quantifies the number of noise experiments (reported random foci throughout the brain) that changes the ALE statistical thresholds such that the significant cluster is no longer significant (Acar et al., 2018). To calculate the FSN, noise experiments with similar sample size and number of foci as the original experiments were generated using an R script (available at https://github.com/NeuroStat/GenerateNull) (Acar et al., 2018). The noise experiments were added to the original experiments and ALE analyses were re-run. Given the minimum amount of injected noise validated for behavioral studies (5k + 10) is predicted to be too high for fMRI meta-analysis, we followed the cut-offs proposed by Cauda et al., 2020, i.e., k/2 as the lower bound and 3k as the upper-bound.

## Results

### Included studies

**Fig. 1:**
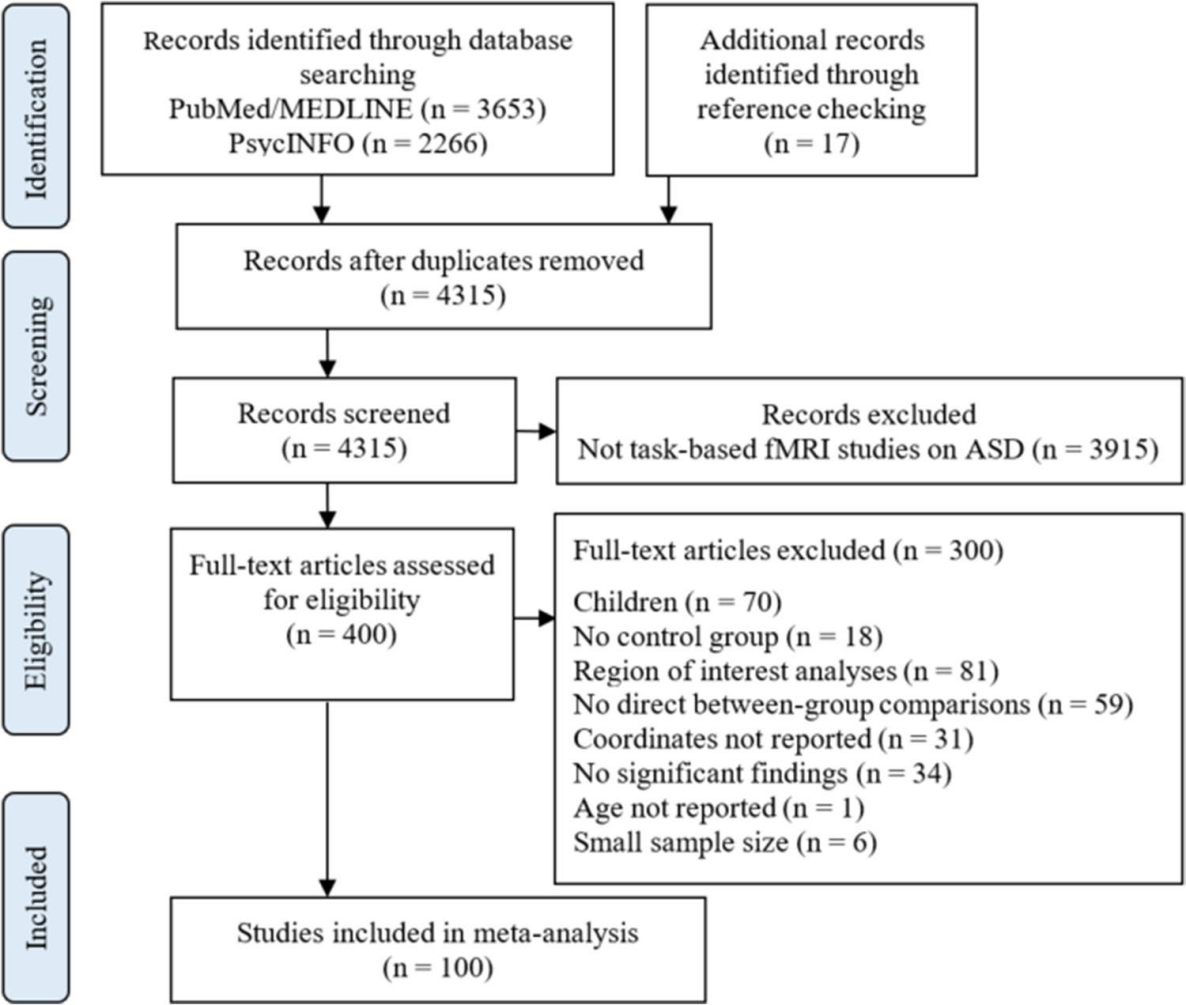
PRISMA flow chart of study screening and selection. ASD, autism spectrum disorders; fMRI, functional magnetic resonance imaging

A total of 4315 articles were identified (after removing duplicates), and 3915 articles were excluded based on title/abstract screen. The full text of 400 articles were screened, with almost perfect agreement between the two authors (percent agreement = 97.25%, Cohen’s kappa = 0.93; McHugh, 2012). A final total of 100 studies were included in the current meta-analysis (Fig. 1). Of these included studies, 93 were from database search and 7 were from additional reference lists and other sources. The final sample included 1679 participants with ASD (range of mean age = 18–41 years, 87% male) and 1717 NC participants (range of mean age = 18–39 years, 85% male). The diagnosis of ASD was confirmed by Autism Diagnostic Observation Schedule (ADOS) and/or Autism Diagnostic Interview-Revised (ADI-R) in 78 studies. The studies were also categorized by task type: there were 57 social studies (e.g., face viewing, emotion recognition, causal attribution) and 43 non-social studies (e.g., visuomotor learning, incentive delay, response inhibition). Supplementary Table 1 shows the participant demographics for each included study, including sample size, age, sex, and IQ for both ASD and NC groups, and diagnosis and diagnostic measure for the ASD group. Supplementary Table 2 shows the description of the experiments for each included study, including fMRI task and stimuli, significant contrast(s), number of foci, reference space, correction for multiple comparisons, and source of coordinates.

### Activation likelihood estimation (ALE)

**Fig. 2:**
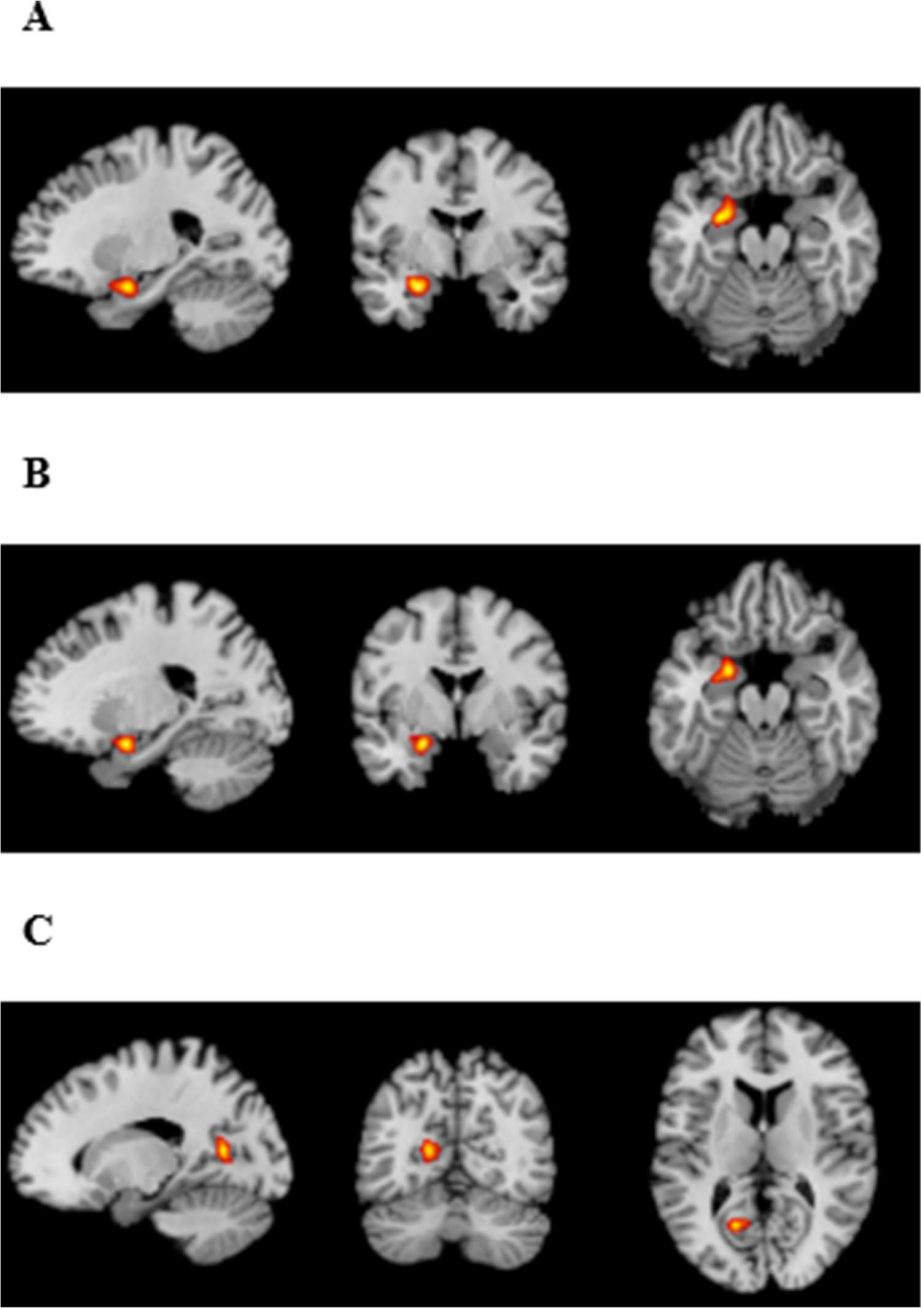
**a** represents results from activation likelihood estimation (ALE) across all tasks combined, showing hypo-activation in the left amygdala in autism spectrum disorder (ASD). **b** represents ALE results for social tasks only, showing hypo-activation in the left amygdala in ASD. **c** represents ALE results for social tasks only, showing hyper-activation in the left posterior cingulate cortex. All results are significant at *p* < .05, cluster-level family wise error (FWE) corrected

Across all studies (n = 100), there were 82 experiments (1358 subjects, 1002 foci, 41 out-of-mask foci) for hypo-activation (ASD < NC) analysis and 56 experiments (851 subjects, 594 foci, 15 out-of-mask foci) for hyper-activation (ASD > NC) analysis. For the hypo-activation analysis, the ASD group (compared to the NC group) showed significantly reduced activation in one cluster in the left amygdala (peak − 26, -2, -20, volume = 1336 mm^3^, maximum ALE value = 0.0327; see Fig. 2a). Ten foci (from nine studies) contributed to this cluster. The tasks used in these studies included: face viewing/affect recognition, moral reasoning, incentive delay, cognitive reappraisal, go/no-go, visual target detection, and sustained attention. For the hyper-activation analysis, no clusters were found.

For social studies (n = 57), there were 50 experiments (841 subjects, 526 foci, 16 out-of-mask foci) for hypo-activation analysis and 28 experiments (451 subjects, 297 foci, 1 out-of-mask focus) for hyper-activation analysis. For the hypo-activation analysis, the ASD group (compared to the NC group) showed significantly reduced activation in one cluster in the left amygdala (peak − 22, 0, -18, volume = 1224 mm^3^, maximum ALE value = 0.023; see Fig. 2b). Seven foci (from six studies) contributed to this cluster. There was great spatial overlap between this cluster and the hypo-activated cluster across all studies, as six foci (from five studies) contributed to both clusters. For the hyper-activation analysis, the ASD group (compared to the NC group) showed significantly increased activation in one cluster in the left posterior cingulate (PCC, BA30, peak − 18, -62, 12, volume = 800 mm^3^, maximum ALE value = 0.018; see Fig. 2c). Five foci (from four studies) contributed to this cluster. For non-social studies (n = 43), there were 32 experiments (517 subjects, 476 foci, 25 out-of-mask foci) for hypo-activation analysis and 28 experiments (400 subjects, 297 foci, 14 out-of-mask foci) for hyper-activation analysis. No clusters were found for either of these analyses.

### Meta-analytic connectivity modelling (MACM)

**Fig. 3:**
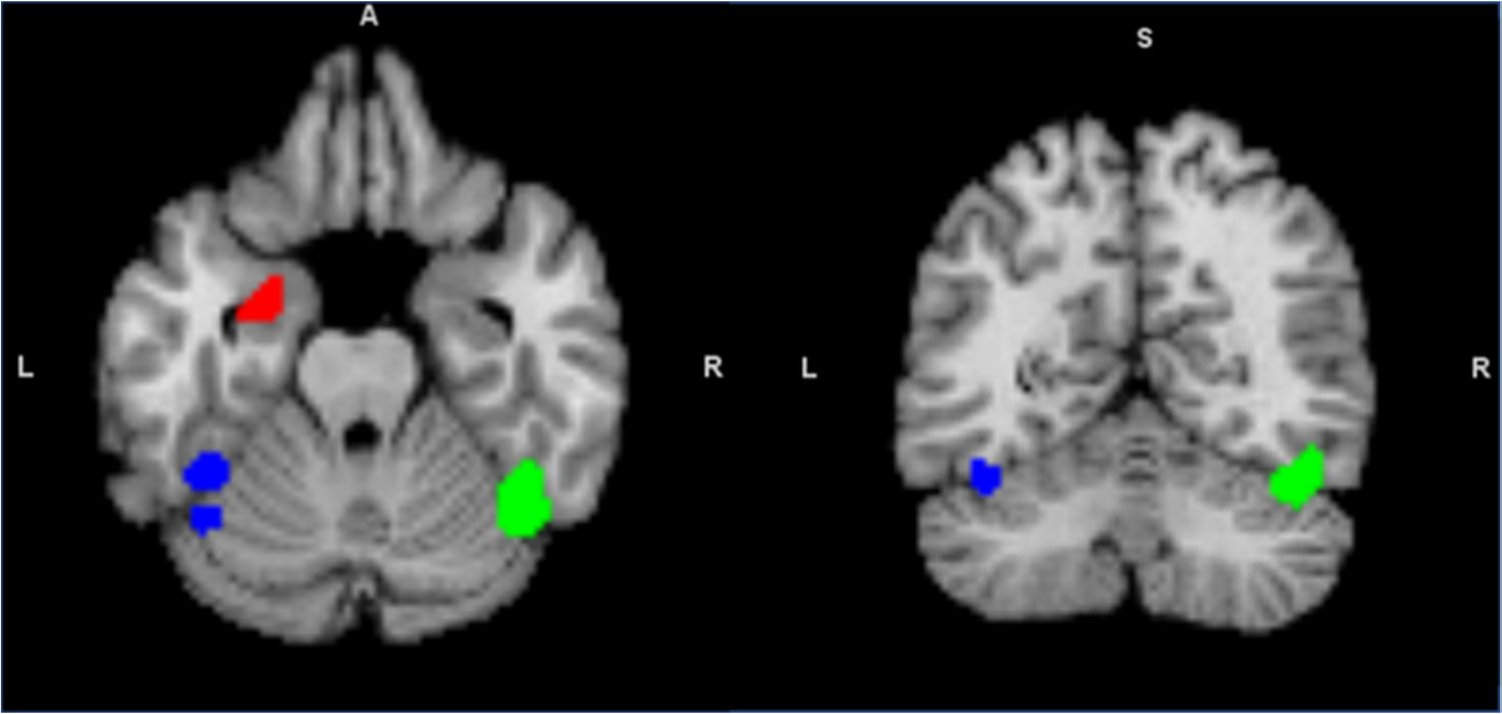
Activation likelihood estimation (ALE) seed region and co-activated clusters from meta-analytic connectivity modelling (MACM). The left amygdala seed region (red) was co-activated with the right cerebellum Lobule VI/Crus I (green) and the left fusiform gyrus/cerebellum Lobule VI/Crus I (blue)

Given that left amygdala was evident in two hypo-activation ALE analyses (all tasks combined and social tasks only), MACM was conducted using this cluster (from all tasks combined analysis) as a seed region. BrainMap search identified 124 experiments (1905 subjects, 1686 foci, 51 out-of-mask foci) that reported at least one focus of activation within the seed. The left amygdala seed (Cluster 1) showed significant co-activations with two clusters (Clusters 2–3) around the right cerebellum (Peak 42, -56, -22, volume = 2560 mm^3^, maximum ALE value = 0.049) and the left fusiform gyrus (FG) (Peak − 42, -46, -18, volume = 1616 mm^3^, maximum ALE value = 0.046)/left cerebellum (Peak − 42, -58, -20, volume = 1616 mm^3^, maximum ALE value = 0.033) (Table 1). The cerebellum regions clustered around the right and left Lobule VI and Crus I (Schmahmann et al., 1999), while the left FG clustered around the left fusiform face area (FFA; Fig. 3).

**Table 1:** Clusters and peak coordinates from MACM analysis

Cluster	Volume (mm^3^)	Label	MNI Coordinates			ALE^†^
			x	y	z	
1	34544	Left Amygdala	-24	-2	-18	0.381
		Right Amygdala	24	-2	-18	0.153
		Left Hypothalamus	-4	-4	-12	0.052
		Left Medial Dorsal Nucleus	0	-14	2	0.051
		Right Claustrum	40	6	-14	0.046
		Right Claustrum	40	-8	-12	0.045
		Left Substantia Nigra	-16	-20	-10	0.039
		Right Insula	46	-16	0	0.032
2	2560	Right Cerebellum	42	-56	-22	0.049
3	1616	Left Fusiform Gyrus (BA 37)	-42	-46	-18	0.046
		Left Cerebellum	-42	-58	-20	0.033

^†^ALE = activation likelihood estimation value

### Behavioral/paradigm analysis

The hypo-activated left amygdala cluster in the ASD group (all tasks combined) was associated with negative emotion (specifically fear, z = 3.047) and face monitor/discrimination paradigm (z = 3.376). The hypo-activated left amygdala cluster (social tasks only) was associated with face monitor/discrimination paradigm (z = 3.323), whereas the hyper-activated left PCC cluster (social tasks only) was not associated with any behavioral domain or paradigm class. While the left FG/cerebellum Lobule VI/Crus I cluster (co-activated with the left amygdala cluster) was associated with language semantics (z = 3.724) and action observation (z = 3.077), the right cerebellum Lobule VI/Crus I cluster (co-activated with the left amygdala cluster) was not associated with any behavioral domain or paradigm class.

### Fail-safe N (FSN)

For the hypo-activated cluster (all tasks combined), noise simulation revealed a total of 22 studies (27%) to be injected into the ALE to produce failure of convergence. For the hypo- and hyper-activated clusters (social tasks only), the number of injected studies was 19 and 15, respectively.

## Discussion

### Summary

This meta-analysis investigated whether hyper- and hypo-activated regions persist in individuals with ASD, irrespective of the task type. Subsequently, we aimed to examine the task-based functional connectivity of the affected regions. Our findings demonstrated the ASD group showed consistent hypo-activation in the left amygdala across all tasks combined. In subgroup analyses investigating social and non-social tasks separately, the left amygdala remained hypo-activated during social but not non-social tasks, while the left PCC showed hyper-activation during social tasks only. Connectivity modelling revealed that in the neurotypical population, the left amygdala cluster is functionally co-activated with the right cerebellum Lobule VI/Crus I and the left FG/cerebellum Lobule VI/Crus I. Behavioral analyses revealed that the left amygdala was associated with negative emotion, and both the left amygdala and PCC were associated with face monitoring/discrimination. The left FG/cerebellum Lobule VI/Crus I cluster was associated with language semantics and action observation. Given that amygdala hypo-activation was stable irrespective of the task stimuli, our findings highlight a common region of aberrant activation which could be used as a future biomarker region for changes in intervention-induced brain activations. Our MACM findings also suggest a role for amygdala-fusiform/cerebellar connectivity related to social cognition in ASD, which should be explored in future research. Examining this connectivity in future research has potential to provide an opportunity for non-invasive brain stimulation tools to target more superficial parts of a network (e.g., the cerebellum) as regions of the limbic system cannot easily be reached via techniques such as repetitive transcranial magnetic stimulation and transcranial direct-current stimulation.

### Aberrant hypo and hyper-activations in ASD

Given the heterogeneous tasks included in our analyses, we do not expect to find as many hypo- or hyper-activated regions compared with previous task-specific meta-analyses, due to the fact that ALE tests for spatial convergence and different tasks involve different regions that may or may not overlap spatially. Indeed, the left amygdala was the only cluster found to be hypo-activated in ASD across all tasks combined, providing strong support for the amygdala theory in ASD (Baron-Cohen et al., 2000). However, it is important to note that three quarters of the studies contributing to this cluster used social tasks, and most of these studies used facial stimuli. Moreover, this cluster remained hypo-activated for social (but not non-social) subgroup analysis and was associated with negative emotion and fear behavioral domains as well as face monitor/discrimination paradigm class. It has previously been suggested that differences in social brain activations between ASD and NC could be contributed by a lack of preference towards social stimuli (Philip et al., 2012), which cannot be ruled out in our meta-analysis. These findings are in line with previous ASD functional neuroimaging meta-analyses, which have reported the left amygdala as one of the differentially activated regions during emotion and face processing (Aoki et al., 2015; Costa et al., 2021) and social cognition (Patriquin et al., 2016).

While aberrant task-based activations of the amygdala are consistently reported within the ASD literature (Kilroy et al., 2019), inconsistencies around whether and when it is hypo- or hyper-activated are in part due to the nature of the task stimuli. For example, within the context of socio-emotional stimuli, it has been shown that amygdala hypo-activation may be more commonly associated with tasks requiring face processing, particularly negative expressions such as fear (Kleinhans et al., 2011), while hyper-activation has been shown during eye gaze aversion (Tottenham et al., 2014). This supports our current findings, given most of the contributing studies to the amygdala cluster used task stimuli that probed a response to facial expressions. Interestingly, we also only observed amygdala hypo-activation unilaterally in the left hemisphere. Previous research has demonstrated amygdala laterality in social cognition, with the left amygdala being suggested to be more sensitive to physiological responses associated specifically with fear (Hardee et al., 2008). This is also in line with our behavioral analysis findings, as the left amygdala cluster was only associated with negative emotion fear and not with other emotion domains.

Aberrant activations of the amygdala, particularly in the adult ASD population, are also in support of the findings from structural neuroimaging studies. These studies have shown enlargement of the amygdala in children, followed by a progressive loss of amygdala neurons into adulthood compared with neurotypical controls (Avino et al., 2018). Whilst the behavioral consequences of such amygdala degeneration in adults remain unclear, direct structure-function relationships between amygdala enlargement and socio-communicative impairments have been observed in toddlers with ASD (Schumann et al., 2009).

In addition to hypo-activation of the amygdala, we also observed hyper-activation of the left PCC, which was associated with the face monitoring/discrimination paradigm. Whilst the PCC cluster only emerged in the subgroup analysis employing social tasks, it is possible that the smaller number of non-social task paradigms (e.g., the go/no-go and visual target detection tasks) that contributed to the hypo-activated amygdala cluster added too much noise and uncertainty for the PCC to be detected in this cluster. However, when only social studies were analyzed, the PCC was found to be hyper-activated in ASD compared to NC, rather than hypo-activated. While the reasons for this are not entirely clear, the PCC has been found to be differently activated in ASD. Previous functional meta-analyses have reported PCC hyper-activation during auditory and language processing in adults (Philip et al., 2012), however hypo-activation was reported during social processing (Di Martino et al., 2009). These differences could be in part due to the classification of social and non-social tasks in different studies, as many auditory and language tasks use human tone and comprehension stimuli, which integrate sensory and social processing required for socio-communicative behaviors (Thye et al., 2018). The PCC is also known as a core functional hub within the default mode network (DMN), overlapping with many regions involved in social and emotional processing that are implicated in the theory of mind difficulties in ASD (Buckner et al., 2008; Leech et al., 2011). Evidence has also shown reduced GABA_B_ inhibitory receptor density within the PCC in individuals with ASD (Leech & Sharp, 2014), which could provide a mechanism for the hyper-activation observed during fMRI. It is plausible that hyper-activation within the PCC may affect functional connectivity in large scale networks such as the DMN (Leech & Sharp, 2014), and resting-state fMRI studies have linked the DMN to both ASD pathophysiology and symptomology (Leech & Sharp, 2014; Monk et al., 2009). Future research should therefore consider the role of the PCC in network connectivity in ASD.

The current meta-analysis did not find any region of aberrant activation for non-social tasks. This is in contrast with previous reviews which have reported differential activation of fronto-parietal regions during executive functioning tasks (May & Kana, 2020) and general non-social tasks (Di Martino et al., 2009). In addition, previous functional neuroimaging meta-analyses examining social and non-social tasks separately in ASD have identified a larger number of hypo- and hyper-activated clusters compared with our findings (Di Martino et al., 2009; Dickstein et al., 2013). Methodological differences in both the individual studies and meta-analytic algorithms could partly explain these differences. For example, ASD presents as a heterogeneous condition, with different subgroups of the condition reported to have different levels of GABA and glutamate activity (Roberts et al., 2020). As both GABA and glutamate metabolisms strongly affect the task-based fMRI BOLD signals (Reynell & Harris, 2013), the increased number of studies included in our meta-analyses (64 additional studies since 2012) may have added more heterogeneity, causing only the most stable regions of aberrant activation to survive thresholding. Second, we used an updated ALE algorithm, which had addressed previous implementation errors in multiple comparison corrections (Eickhoff et al., 2017). Third, we applied cluster-level FWE correction (instead of false-discovery rate correction) and followed the recommended more conservative thresholds (Eickhoff et al., 2016; Müller et al., 2018). Importantly, our findings suggest that key social and DMN regions including the amygdala and the PCC could serve as brain biomarkers to assess brain-behavioural responses to future interventional research, given their consistent aberrant activations across the neuroimaging literature.

### Meta-analytic connectivity modelling of the amygdala seed region

In the neurotypical population, the amygdala is a widely connected region, with different subregions shown to co-activate with different brain areas (Bzdok et al., 2013). In this meta-analysis, the cluster around the left amygdala (seed region for hypo-activation) co-activated with the right cerebellum Lobule VI/Crus I and the left FG/cerebellum Lobule VI/Crus I. Behavioral analyses also showed that the left FG/cerebellum Lobule VI/Crus I was associated with language semantics and action observation. These findings are supported by large neuroimaging reviews in healthy individuals that have implicated Crus I, Lobules IV and VI in social cognition and emotion processing (Keren‐Happuch et al., 2014; Stoodley, 2016; Van Overwalle et al., 2014, 2020).

FFA hypo-activation has consistently been reported in ASD (Schultz, 2005), and previous ASD functional meta-analyses have reported the left FG as one of the hypo- and/or hyper-activated regions during face (Nickl-Jockschat et al., 2015), visual (Samson et al., 2012), language (Herringshaw et al., 2016), and social processing tasks separately (Di Martino et al., 2009; Philip et al., 2012). The involvement of the cerebellum in many ASD symptoms has become increasingly recognized in recent years (Becker & Stoodley, 2013; Hampson & Blatt, 2015; Rogers et al., 2013), and previous ASD functional meta-analyses have also reported the cerebellum as one of the hypo-activated regions during social processing (left cerebellum culmen in adults, Dickstein et al., 2013; right cerebellum culmen, Philip et al., 2012), reward processing (left and right cerebellum, Clements et al., 2018), action observation (right cerebellum Lobule VI, Chan & Han, 2020), social/pragmatic/auditory language processing and sentence comprehension (right cerebellum, Herringshaw et al., 2016).

Task-based functional connectivity measured by MACM could complement findings from resting-state functional connectivity studies (Laird et al., 2013). Resting-state functional connectivity studies on adolescents and adults with ASD have found weaker left amygdala connectivity with right thalamus, right putamen, left occipital pole, and biliteral lateral occipital cortex, and weaker right amygdala connectivity with left thalamus and right superior parietal lobule (Guo et al., 2016; Rausch et al., 2016). Different amygdala subregions have also showed weaker or stronger resting-state functional connectivity with different brain regions in adolescents and adults with ASD (Kleinhans et al., 2016; Rausch et al., 2016), and some disrupted amygdala connectivity has been linked to ASD social symptoms (Guo et al., 2016; Kleinhans et al., 2016).

The left cerebellum was one of the regions that showed weaker resting-state functional connectivity with both left and right amygdala in pre-school children with ASD (Shen et al., 2016). Disrupted resting-state functional connectivity between amygdala and cerebellum has also been reported in adolescents with generalized anxiety disorder (Roy et al., 2013), adults with major depressive disorder (Ramasubbu et al., 2014; Tang et al., 2019), and children with attention-deficit/hyperactivity disorder (ADHD) (Yu et al., 2020). Whilst studies looking specifically at ASD with comorbid conditions were excluded in our meta-analyses, the potential effects of anxiety, depression and/or undiagnosed ADHD on fMRI responses to socio-emotional stimuli in ASD cannot be ruled out.

The amygdala-fusiform system has also been suggested to be implicated in ASD deficits in face perception and social cognition (Dziobek et al., 2010; Schultz, 2005). The left amygdala was one of the regions that showed weaker functional connectivity with the right fusiform face area in adults with ASD during face identification, and this weaker connectivity was related to social impairment (Kleinhans et al., 2008). While ASD studies have not found disrupted resting-state functional connectivity between left amygdala and left FG to date, this connectivity has been reported to be disrupted in adults with social anxiety disorder (Jung et al., 2018), suggesting the need for future research in adults with ASD, and particularly those with comorbid social cognitive and anxiety symptoms.

### Limitations

This meta-analysis highlights consistent regions of aberrant functional activations that can form the focus of adult ASD interventions. While a large number of studies were included, the generalizability of the findings is limited by the participant group in the individual studies. Most studies continue to recruit mostly male participants, likely due to the underdiagnosis and differences in symptom presentation in females with ASD. Most subjects recruited were also high functioning, reflecting the selection bias against individuals with comorbid intellectual disability in the broader ASD literature (Russell et al., 2019). Nevertheless, high-functioning adults with ASD remain an important population for research, as they may often be overlooked for learning and behavioral interventions. Since the number of studies reporting age-ranges was too small to allow for reliable screening, we chose to use the mean age (≥ 18 years) to focus on an adult population, as previous meta-analyses of this nature have also used this method (Philip et al., 2012). However, it is important to note that this does not preclude the inclusion of adolescents and children into some of these studies. It is encouraged that future work report the age-range and mean of their data to facilitate a greater understanding of age-related differences in brain structure and function in ASD. Another limitation is that currently there are no clear cut-offs for publication bias in neuroimaging meta-analyses to date, and disease studies in general appear to be less robust against noise studies (Samartsidis et al., 2020). Thus, it will be important for future research to examine the optimal methods for addressing publication bias in neuroimaging meta-analyses to draw stronger conclusions.

## Conclusion

This meta-analysis highlights key social and DMN regions including the amygdala and PCC to be consistently differentially activated in ASD. The fusiform gyrus and cerebellum lobule VI/Crus I could also play a role in ASD social cognitive deficits through functional connections with the amygdala. Future research should consider fusiform/cerebellar involvement with limbic structures and how this connectivity relates to social and non-social cognition in ASD.

## Supplementary information

Below is the link to the electronic supplementary material.

ESM 1 (DOCX 75.5 KB)

## Data Availability

All included studies identified through PubMed and PsychInfo were coded using Scribe, and submitted to BrainMap, where they were quality checked and added to the BrainMap database corpus. The data that support the findings of this study including input data in GingerALE-compatible format are available in the NIE Data Repository 10.25340/R4/IWIXGE for replication and/or extension of findings.

## Code availability

These data were derived from the following software applications available in the public domain: BrainMap database via Sleuth https://www.brainmap.org/software.html#Sleuth. GingerALE software was used to run ALE meta-analysis and meta-analytic connectivity modelling (MACM) and is available at https://www.brainmap.org/ale/. MANGO application with behavioral and paradigm class plugin was used to run behavioral analysis and can be found at http://ric.uthscsa.edu/mango/. Code for fail safe is publicly available from https://github.com/NeuroStat/GenerateNull.
