# Supplementary material for: Meta-analytic connectivity modelling of functional magnetic resonance imaging studies in autism spectrum disorders
Source: Brain Imaging Behav. 2023 Jan 12;17(2):257–69. doi: 10.1007/s11682-022-00754-2 (PMC10049951; doi:10.1007/s11682-022-00754-2)
Supplement: Supplementary file 1 — (DOCX 75.5 KB) [file 11682_2022_754_MOESM1_ESM.docx]

**APPENDICES**

Supplementary Table 1

*Participant demographics for each included study*

| **Study** | **ASD** | | | | | **Control** | | |
| --- | --- | --- | --- | --- | --- | --- | --- | --- |
|  | **N (M:F)** | **Age Mean (SD)** | **FSIQ Mean (SD)** | **Diagnosis** | **Diagnostic Measures** | **N (M:F)** | **Age Mean (SD)** | **FSIQ Mean (SD)** |
| Alaerts et al. (2014) | 15:0 | 21.7 (4) | 107.9 (13.9) | autistic disorder (DSM-IV-TR) | NA | 15:0 | 23.3 (2.9) | 114.8 (12.8) |
| Ammons et al. (2018) | 10:4 | 25.07 (6.16) | 117.57 (12.97) | ASD | ADI, ADOS | 13:1 | 24.86 (5.35) | 117.23 (8.78) |
| Anderson et al. (2010) | 26:0 | 21.7 (6.4) | PIQ= 102.8 (16.7) | autism (DSM-IV, ICD-10) | ADI-R, ADOS-G | 15:0 | 22.5 (6.3) | PIQ= 116.3 (16.4) |
| Aoki et al. (2014) | 17:0 | 29.6 (8) | 106.7 (12) | 19 HFA, 1 Asperger (DSM-IV-TR) | ADI-R, ADOS | 17:0 | 30.4 (5.6) | 108.7 (7.6) |
| Barbeau et al. (2015) | 19:3 | 20.3 (5.5) | 98.6 (10.7) | autism (DSM-IV) with speech onset delays/atypicalities | ADI-R, ADOS-G | 21:3 | 22.7 (5.3) | 108.1 (13) |
| Beacher et al. (2012) | 15:14 | 32.8 (9.1) | NART= 30.3 (7.56) | Asperger (DSM-IV-TR) | DISCO | 16:16 | 30.4 (7.7) | NART= 35.2 (6.17) |
| Bird et al. (2010) | 18:0 | 34.6 (13.3) | 115.8 (14.6) | 15 Asperger, 3 autism (DSM-IV) | ADOS-G | 18:0 | 35 (12.8) | 118.8 (11.7) |
| Bolte et al. (2015) | 30:2 | 19.3 (14-33) | NVIQ= 105.7 (12) | 14 Asperger, 10 autism, 8 atypical autism/PDD-NOS (ICD-10) | ADI-R, ADOS | 21:4 | 19.7 (14-27) | 109 (12.2) |
| Caria et al. (2011) | 6:2 | 23.4 (7.03) | NA | Asperger (DSM-IV, ICD-10) | ADOS-G, GADS, KADI | 6:8 | 24.3 (3.02) | NA |
| Cascio et al. (2012) | 12:1 | 28.3 (10.7) | 103.2 (20.7) | ASD | ADI-R, ADOS | 14:0 | 30.8 (12) | 107.9 (12.9) |
| Choiunard et al. (2017) | 9:3 | 32.4 (10.8) | NA | ASD | ADOS-2 | 8:4 | 33 (10.1) | NA |
| Ciaramidaro et al. (2018) | 31:2 | 18.76 (4.98) | 105.82 (13.75) | 15 Asperger, 10 autism, 8 atypical autism (ICD-10) | ADI-R, ADOS | 21:4 | 19.68 (3.45) | 109 (12.55) |
| Cooper et al. (2017) | 11:9 | 30.9 (8.9) | NA | ASD (DSM-V, ICD-10) | NA | 11:9 | 29.8 (6.7) | NA |
| Corradi-Dell’Acqua et al. (2014) | 10:0 | 21.5 | 100.9 | 6 Asperger, 4 HFA | ADI-R, ADOS-G | 10:0 | 21.55 | 109.5 |
| Critchley et al. (2000) | 9:0 | 37 (7) | 102 (15) | 7 Asperger, 2 autism (ICD-10) | ADI | 9:0 | 27 (7) | 116 (10) |
| Cygan et al. (2019) | 15:0 | 24.33 | NA | ASD | ADOS | 15:0 | 24.17 | NA |
| Damarla et al. (2010) | 11:2 | 19 (5.5) | 109.5 (8.7) | autism | ADI-R, ADOS-G | 13:0 | 22.1 (4.25) | 111.5 (5.75) |
| Deeley et al. (2007) | 9:0 | 34 (10) | 114 (12) | Asperger (DSM-IV, ICD-10) | ADI, ADOS | 9:0 | 27 (5) | 120 (18) |
| Dichter et al. (2009) | 14:1 | 23.3 (11.1) | 102.8 (24) | ASD | ADI-R, ADOS-G | 18:1 | 28 (7.9) | 114 (9.4) |
| Dichter et al. (2012) | 15:0 | 30.1 (11.6) | 111.9 (22.7) | 13 HFA, 2 Asperger | ADOS-G | 16:0 | 27.5 (7.5) | 122.2 (10.7) |
| Dichter et al. (2012) | 14:2 | 26 (9.1) | 109.9 (20.3) | 14 HFA, 2 Asperger | ADOS-G | 14:6 | 25.4 (7) | 127 (8.1) |
| Duerden et al. (2013) | 14:5 | 26.8 (5.7) | 111.89 (13.71) | ASD (DSM-IV) | ADI-R, ADOS-G | 15:5 | 33.7 (9.6) | 114.32 (14.8) |
| Ewbank et al. (2017) | 10:5 | 31.8 (9.2) | 126 (11.8) | autism, Asperger (DSM-IV) | ADI-R, ADOS, AAA | 9:6 | 28.1 (7.5) | 128.2 (10.5) |
| Fan et al. (2012) | 9:3 | 30 (6) | 115 (14) | 8 autism, 4 Asperger (DSM-IV-TR) | ADI-R, ADOS-G | 10:2 | 28 (7) | 120 (15) |
| Fan et al. (2014) | 24:0 | 18.4 (2.8) | 107 (11.2) | ASD (DSM-IV) | ADI-R | 21:0 | 19.3 (3.4) | 111.5 (10.3) |
| Gadgil et al. (2013) | 14:3 | 31.8 (12) | 109.6 (12.1) | 9 autistic disorder, 8 Asperger (DSM-IV) | ADOS-G | 13:3 | 34.8 (11.4) | 116.7 (18.1) |
| Gaffrey et al. (2007) | 10 | 26.1 (10.5) | 101.5 (11.9) | 8 autism, 2 Asperger (DSM-IV) | ADI-R, ADOS | 10 | 25.3 (9.8) | 112.6 (12.6) |
| Gebauer et al. (2014) | 17:2 | 26.16 (5.63) | 108.32 (14.56) | ASD | ADOS-G | 18:2 | 24.45 (4.57) | 114.5 (12.37) |
| Georgescu et al. (2013) | 9:4 | 31.23 (4.87) | VIQ= 108.46 (8.1) | Asperger/HFA (ICD-10) | clinical interview, neuropsychological assessment | 9:4 | 30.23 (3) | VIQ= 108.92 (9.23) |
| Gilbert et al. (2008) | 12:3 | 38 (13) | 119 (14) | ASD | ADOS-G | 13:5 | 32 (8) | 119 (11) |
| Grezes et al. (2009) | 10:2 | 26.6 (10.4) | 102 (20.6) | 10 Asperger, 2 HFA (DSM-IV) | NA | 12:0 | 21 (1.6) | 119 (6.6) |
| Gu et al. (2015) | 15:0 | 26.2 (6.4) | 109.5 (18) | 12 autism disorder, 5 Asperger (DSM-IV-TR) | ADI-R, ADOS-G | 15:0 | 26.8 (7.8) | 113.5 (11.9) |
| Hadjikhani et al. (2014) | 33:3 | 23.5 (8.7) | 107.4 (15.8) | 24 Asperger, 10 autistic disorder, 2 PDD-NOS (DSM-IV-TR) | ADI-R, ADOS, DSCD | 28:3 | 22.5 (7.5) | 112.9 (10.7) |
| Haist et al. (2005) | 8:0 | 23.4 (11.4) | 101 (9.3) | 6 autistic disorder, 2 Asperger (DSM-IV) | ADI-R, ADOS | 8:0 | 25.6 (3.8) | 112.2 (8.8) |
| Hames et al. (2016) | 7:3 | range=20-28 | NA | ASD | various means and testing | 3:8 | range=20-28 | NA |
| Herrington et al. (2007) | 10:0 | 27.6 (7.1) | 109 | Asperger (DSM-IV, ICD-10) | mental health professionals | 10:0 | 25.6 (4.8) | 119 |
| Hesling et al. (2010) | 8:0 | 23.38 (2.1) | VIQ= 89 (7.89) | HFA (DSM-IV-R) | ADI-R | 8:0 | 23.05 (2.02) | VIQ= 128.33 (4.58) |
| Hsu et al. (2018) | 15:11 | 35.08 (2.24) | Ravens= 55.5 (5) | ASD (DSM-IV) | ADOS | 18:12 | 30.73 (2.09) | Ravens= 48.5 (4.65) |
| Ishitobi et al. (2011) | 8:1 | 23.2 (6.9) | 110.7 (8.4) | 6 Asperger, 3 HFA (DSM-IV) | DISCO | 12:12 | 23.1 (4.4) | 112.7 (20.1) |
| Kana et al. (2006) | 11:1 | 22.5 (8.8) | 110.7 (9.2) | autism | ADI-R, ADOS-G | 12:1 | 20.3 (4) | 113.2 (9.2) |
| Kana et al. (2007) | 11:1 | 26.8 (7.7) | 110.1 (12.6) | autism | ADI-R, ADOS-G | 11:1 | 22.5 (3.2) | 117 (8.7) |
| Kana & Wadsworth (2012) | 16:0 | 20 (6.43) | 130.8 (16.84) | ASD | ADI-R, ADOS-G | 16:0 | 21.6 (2.7) | 113.44 (9.18) |
| Kana et al. (2014) | 15 | 21.14 (0.99) | 106.93 (4.84) | ASD | ADI-R, ADOS | 15 | 22.28 (1.08) | 112 (2.24) |
| Kana et al. (2017) | 15:0 | 21.63 (5.54) | 104 (13.76) | ASD | ADI-R, ADOS | 15:0 | 27.25 (5.19) | 114.29 (7.01) |
| Kennedy et al. (2006) | 12:0 | 25.49 (9.61) | 96.1 (16.5) | 10 HFA, 3 Asperger, 2 PDD-NOS | ADI-R, ADOS | 14:0 | 26.07 (7.95) | NA |
| Kennedy et al. (2008) | 13:0 | 26.9 (12.3) | 101.7 (14.6) | 6 autism, 6 Asperger, 1 PDD-NOS | ADI-R, ADOS | 12:0 | 27.5 (10.9) | 111.5 (8.3) |
| Kestemont et al. (2016) | 9:3 | 30.17 | NA | ASD | clinical diagnosis | 7:13 | 23.4 | NA |
| Kleinhans et al. (2008) | 14:0 | 23.79 (9.58) | 98.14 (11.84) | 8 autistic disorder, 3 Asperger, 3 PDD-NOS (DSM-IV) | ADI-R, ADOS-G | 14:0 | 22.41 (8.67) | 113.43 (12.91) |
| Kleinhans et al. (2011) | 28 | 23.57 (6.6) | 113.3 (14.22) | 15 Asperger, 11 autistic disorder, 2 PDD-NOS (DSM-IV) | ADI-R, ADOS | 25 | 23.32 (5.15) | 112.05 (15.17) |
| Kleinhans et al. (2016) | 25:2 | 23.57 (6.6) | 110.81 (15.68) | 14 Asperger, 11 autistic disorder, 2 PDD-NOS (DSM-IV) | ADI-R, ADOS | 23:2 | 23.32 (5.15) | 112.92 (12.29) |
| Koshino et al. (2008) | 11:0 | 24.5 (10.2) | 104.5 (13.1) | autism | ADI-R, ADOS-G | 10:1 | 28.7 (10.9) | 108.6 (9.1) |
| Lassalle et al. (2017) | 27:0 | 23.63 (9.86) | 113.15 (12.36) | ASD (DSM-IV-TR) | ADI-R, ADOS, DISCD | 21:0 | 19.7 (7.74) | 112 (13.73) |
| Lassalle et al. (2019) | 17:2 | 25.27 (8.83) | 111.21 (8.83) | 11 Asperger, 6 ASD, 2 PDD-NOS (DSM-IV-TR) | ADI-R, ADOS | 17:3 | 24.15 (7.57) | 111.85 (9.15) |
| Marsh et al. (2011) | 18 | 33 (10.9) | PIQ= 104.4 (18) | 13 Asperger, 5 HFA | ADOS | 19 | 32.2 (10.1) | PIQ= 113.4 (13.9) |
| Martineau et al. (2010) | 7:0 | range=19-31 | range= 85-113 | ASD | NA | 8:0 | range=19-31 | NA |
| Mizuno et al. (2011) | 14:1 | 24.7 (7.8) | 106.3 (10.7) | autism | ADI-R, ADOS | 15:0 | 24.7 (7.7) | 108.7 (5.1) |
| Morita et al. (2012) | 14:1 | 23.7 (4.3) | 105.4 (11.7) | 10 Asperger, 5 autistic disorder (DSM-IV-TR) | DISCO | 13:2 | 23.3 (3.6) | 110.1 (4.3) |
| Morita et al. (2016) | 14:0 | 24.5 (6.7) | 109.8 (13.9) | 13 autistic disorder, 1 Asperger (DSM-IV-TR) | DISCO | 18:0 | 23.3 (3.3) | 108.1 (8) |
| Moseley et al. (2015) | 18 | 30.4 (10) | 113.5 (23) | 17 Asperger, 1 PDD-NOS (DSM-IV) | AQ | 18 | 28.6 (11.7) | 110.2 (12.3) |
| Muller et al. (2001) | 8:0 | 28.4 (8.9) | 86.5 (11.4) | autism (DSM-IV) | ADI-R, CARS | 8:0 | 28.5 (21-43) | NA |
| Muller et al. (2003) | 8:0 | 28.4 (8.9) | 86.5 (11.4) | autism (DSM-IV) | ADI-R, CARS | 8:0 | range=21-43 | NA |
| Muller et al. (2004) | 8:0 | 28.4 (8.9) | 86.5 (11.4) | autism (DSM-IV) | ADI-R, CARS | 8:0 | range=21-43 | NA |
| Murphy et al. (2014) | 46:0 | 18 (6) | 114 (14) | ASD (32 Asperger, 14 childhood autism; ICD-10) | ADI-R, ADOS | 44:0 | 19 (6) | 117 (12) |
| Murphy et al. (2017) | 38:0 | 18.18 (5.75) | 113 (14) | ASD (28 Asperger, 10 childhood autism; ICD-10) | ADI-R, ADOS | 40:0 | 20.41 (5.5) | 116 (12) |
| Nijhof et al. (2018) | 13:11 | 32.8 (8.4) | 106.4 (16) | ASD | ADOS-2 | 11:10 | 31.1 (8.6) | 114 (9.1) |
| Ohta et al. (2012) | 21:3 | 30.2 (7.6) | 108.7 (12.7) | 12 Asperger, 9 HFA, 3 PDD-NOS (DSM-IV) | interviews | 22:3 | 32.4 (7.5) | NA |
| Okamoto et al. (2018) | 18:0 | 28.2 (6.9) | 109.2 (12.6) | ASD (DSM-V) | DISCO | 18:0 | 24.8 (5) | 113.7 (8.6) |
| Pantelis et al. (2015) | 12:5 | 28.9 (8.9) | 111 (12) | ASD (DSM-IV-TR) | ADI-R, ADOS, SCQ | 18:3 | 26.9 (5.5) | 112 (9) |
| Perkins et al. (2015) | 12:0 | 18.5 (2.5) | NA | autistic disorder, Asperger (DSM-IV) | clinical psychologist | 12:0 | 19.75 (4.93) | NA |
| Perlman et al. (2011) | 11:1 | 25.5 (7.47) | VIQ= 106.7 (11.7) | autism (DSM-IV) | ADI-R, ADOS | 7:0 | 28.57 (5.74) | VIQ= 114.9 (4.4) |
| Poulin-Lord et al. (2014) | 20:3 | 19.8 (4.72) | 100.3 (10.48) | autism (DSM-IV) | ADI-R, ADOS | 19:3 | 22.6 (5.56) | 107.3 (12.51) |
| Redcay et al. (2013) | 10:3 | 28 | CIQ= 122 | ASD | ADOS | 11:3 | 27 | CIQ= 117 |
| Richey et al. (2015) | 13:2 | 26.1 (8.1) | 113.6 (13.8) | ASD (5 Asperger) | ADOS-G | 13:2 | 27.4 (8.3) | 111.4 (11.2) |
| Rosenblau et al. (2017) | 14:6 | 31.8 (9.3) | 113 (17.3) | autism, Asperger (DSM-IV) | ADI-R, ADOS, ASDI | 15:6 | 31.9 (9.3) | 108.3 (13.6) |
| Sabatino et al. (2013) | 13:2 | 26.3 (9.4) | 109.9 (20.3) | 13 HFA, 2 Asperger | ADOS-G | 12:5 | 24.3 (3.7) | 127 (8.1) |
| Samson et al. (2015) | 13:1 | 22.92 (6.58) | 106.8 (16.5) | ASD (DSM-IV) | ADI-R, ADOS-G | 11:2 | 23.5 (7.42) | 109.6 (10.3) |
| Sato et al. (2017) | 15:1 | 26.1 (6.3) | 114.2 (12.4) | 9 PDD-NOS, 7 Asperger (DSM-IV-TR) | CARS | 15:2 | 24 (4.5) | NA |
| Schelinski et al. (2016) | 13:3 | 33.75 (10.12) | 110.31 (13.79) | 14 Asperger, 2 childhood autism (ICD-10) | ADI-R, ADOS | 13:3 | 33.69 (9.58) | 111.5 (10.97) |
| Schipul et al. (2016) | 14:2 | 26.5 (6.4) | 13.8 (12.4) | ASD | ADI-R, ADOS | 14:2 | 25.4 (7.7) | 113.8 (8.5) |
| Schmitz et al. (2006) | 10:0 | 38 (9) | 105 (14) | 8 Asperger, 2 HFA (ICD-10) | ADI | 12:0 | 39 (6) | 106 (13) |
| Schmitz et al. (2008) | 10:0 | range=20-50 | NA | 7 Asperger, 3 HFA (ICD-10) | ADI | 10:0 | range=20-50 | NA |
| Schneider et al. (2013) | 15:13 | 31.39 (8.97) | 109.07 (9.12) | ASD (DSM-IV) | ADOS-G, AQ | 28 | 31.29 (9.03) | 114.04 (9.55) |
| Schulte-Ruther et al. (2011) | 14:0 | 27.4 (9.3) | GIQ= 106.6 (10.5) | 7 Asperger, 7 HFA (DSM-IV, ICD-10) | ADOS, AQ | 14:0 | 25.1 (6.7) | GIQ= 112.1 (10.4) |
| Shafritz et al. (2008) | 16:2 | 22.3 (8.7) | 102.5 (17.6) | HFA (DSM-IV) | ADI-R, ADOS | 13:2 | 24.3 (6.2) | 111.4 (15.1) |
| Shafritz et al. (2015) | 12:3 | 18.1 (13-23) | 101.5 (18.6) | 11 autistic disorder, 4 Asperger (DSM-IV) | ADI-R, ADOS | 12:3 | 18.4 (12-23) | 115.2 (9.3) |
| Silani et al. (2008) | 13:2 | 36.6 (11.7) | 117.6 (13.5) | autism, Asperger (DSM-IV) | ADOS | 13:2 | 33.7 (10.3) | 119.6 (11.4) |
| Simard et al. (2015) | 13:2 | 22.4 (5.95) | 100.87 (12.05) | autism (DSM-IV) | ADI-R, ADOS-G | 15:3 | 21.72 (5.2) | 106.22 (12.97) |
| Solomon et al. (2015) | 18:4 | 22.95 (5.11) | 112.64 (12.44) | 16 Asperger, 5 HFA, 1 PDD-NOS (DSM-IV-TR) | ADOS-G | 20:5 | 23.36 (4.15) | 114.17 (11.51) |
| Sommer et al. (2018) | 10:5 | 28.2 (10.4) | VIQ= 113.5 (10.1) | 11 Asperger, 4 autism (ICD-10) | AAA | 10:5 | 29.9 (12.2) | VIQ= 112.8 (13.9) |
| Soulieres et al. (2009) | 11:1 | 22.08 (4.91) | 101.5 (12.56) | autism (DSM-IV) | ADI-R, ADOS-G | 11:2 | 20.15 (3.02) | 105.31 (14.49) |
| Stanfield et al. (2017) | 19:5 | 40.5 (11.9) | 113.9 (17.1) | autism, Asperger (DSM-IV) | ADOS-G | 22:10 | 36.6 (9.5) | 117.9 (10.0) |
| Takarae et al. (2007) | 13 | 24.5 (7.7) | 105.9 (12.3) | autism (DSM-IV) | ADI-R, ADOS-G | 14 | 26.6 (7.8) | 110.3 (13.7) |
| Tanabe et al. (2012) | 16:5 | 25.1 (5.3) | 101.2 (16.2) | 16 autism, 5 Asperger (DSM-IV-TR) | DISCO | 19:0 | 23.8 (3.5) | NA |
| Tietze et al. (2019) | 13:3 | 39.5 (11.17) | VIQ= 30.28 (4.37) | Asperger (DSM-IV-TR) | self-developed semi-structured interview | 15:1 | 33.75 (8.22) | VIQ= 31.72 (4.1) |
| Travers et al. (2015) | 15:0 | 20.81 (3.98) | 109.47 (16.93) | ASD | ADI-R, ADOS-G | 15:0 | 21.41 (2.85) | 112.73 (8.39) |
| Velasquez et al. (2017) | 13:6 | 25.84 (4.39) | 111.53 (12.82) | ASD (DSM-IV) | ADI-R, ADOS-G | 16:6 | 29.03 (9.4) | 112.27 (11.84) |
| Wicker et al. (2008) | 11:1 | 27 (11) | (59-124) | 8 autism, 4 Asperger (DSM-IV) | screening questionnaire for ASD | 14:0 | 23.4 (10) | NA |
| Yamada et al. (2012) | 22:3 | 30.7 (7.78) | 106.9 (15.9) | 11 Asperger, 11 HFA, 3 PDD-NOS (DSM-IV) | clinical psychologist, psychiatrist | 22:4 | 32.2 (7.7) | 103.9 (11.4) |
| Zurcher et al. (2013) | 13:3 | 23.5 (6.8) | PIQ= 108.7 (13.3) | 7 autism, 7 Asperger, 2 PDD-NOS | ADI-R, ADOS | 16:2 | 25.8 (5.3) | PIQ= 112.1 (9) |
| Zurcher et al. (2013) | 19:3 | 27.6 (7.7) | PIQ= 114 (15) | ASD (DSM-IV-TR) | ADI-R, ADOS, DISCO-10 | 19:3 | 23.7 (5.9) | PIQ= 112 (8) |

NA = not specified

Supplementary Table 2

*Description of experiments for each included study*

| **Study** | **Task** | **Stimuli** | **Social/Non-Social** | **Contrast** | **Number of Foci** | | **Reference Space** | **Multiple Comparison Correction** | **Source of Coordinates** |
| --- | --- | --- | --- | --- | --- | --- | --- | --- | --- |
|  |  |  |  |  | **ASD < TD** | **ASD > TD** |  |  |  |
| Alaerts et al. (2014) | emotion recognition | point-light figures | social | emotion > fixation | 2 | 0 | MNI | no | Table 2 |
| Ammons et al. (2018) | social movement observation | stick figure human characters and geometrical shapes | social | social > random  human > shape | 1 | 1 | MNI | yes | Table s2, s3 |
| Anderson et al. (2010) | auditory and visual language | auditory phrases that described a common word, visual sentences with a blank | non-social | auditory > fixation | 1 | 0 | MNI | yes | Page 135 |
| Aoki et al. (2014) | social emotions and beliefs inference | stories that require understanding of first-order false beliefs | social | social – belief  belief – control | 3 | 0 | MNI | no | Table 3 |
| Barbeau et al. (2015) | visuo-motor (Poffenberger) | a black square appeared to left or right | non-social | RH – fixation  LH – fixation  LVF – fixation  RVF – fixation | 12 | 0 | MNI | yes | Table 2 |
| Beacher et al. (2012) | verbal fluency | letters | non-social | word generation – baseline | 0 | 3 | MNI | yes | Table 1 |
| Bird et al. (2010) | empathy for pain | participant’s and partner’s hands, colored arrow cues of different brightness | social | high pain – low pain: other > self | 1 | 0 | MNI | no | Page 1521 |
| Bolte et al. (2015) | facial affect recognition,  object recognition (control) | pictures of facial/neutral affect,  squares and circles in oval shape (control) | social | implicit > explicit  implicit > control | 16 | 0 | MNI | yes | Table 2 |
| Caria et al. (2011) | music emotional processing | happy and sad music,  random tones (control) | social | happy > control (standard and favorite > baseline)  happy > control (favorite > standard)  sad > control (standard and favorite > baseline)  sad > control (favorite > standard) | 15 | 0 | MNI | yes | Table 5 |
| Cascio et al. (2012) | tactile stimulation | textured surfaces: brush, burlap, mesh | non-social | brush  burlap  mesh | 32 | 5 | MNI | yes ^†^ | Table 3 |
| Choiunard et al. (2017) | auditory sentence decision | literally true, metaphor, literally false, scrambled metaphor sentences | non-social | metaphors > rest | 0 | 6 | MNI | yes | Page 25 |
| Ciaramidaro et al. (2018) | facial affect recognition,  object recognition (control) | angry and fearful faces,  neutral faces and an oval geometric figure (control) | social | implicit FAR > object recognition | 16 | 0 | MNI | yes ^†^ | Table 3 |
| Cooper et al. (2017) | episodic memory | objects with backgrounds | non-social | encoding – baseline  retrieval – baseline | 2 | 0 | MNI | no | Table s1 |
| Corradi-Dell’Acqua et al. (2014) | gender discrimination | hybrid faces | social | HSF > LSF | 5 | 0 | MNI | yes | Table 3 |
| Critchley et al. (2000) | emotion recognition | faces | social | NA | 1 | 2 | Talairach | yes | Table 2 |
| Cygan et al. (2019) | self-reflection | written adjectives | non-social | all conditions (present-self, past-self, close-other)  past-self | 0 | 7 | Talairach | yes | Table 3 |
| Damarla et al. (2010) | embedded figures | objects | non-social | EFT – fixation | 5 | 5 | MNI | no | Table 2 |
| Deeley et al. (2007) | facial emotional processing | faces | social | fear > baseline  disgust > baseline  happy > baseline  sad > baseline  neutral fear  mild fear  intense fear  neutral disgust  mild disgust  intense disgust  mild happy  intense happy  neutral sad  mild sad  intense sad  neutral faces (fear, disgust, happy, sad) | 63 | 7 | Talairach | no | Table 3, s5-s9 |
| Dichter et al. (2009) | social target detection | squares, circles, triangles, neutral faces | social | face > novel  shape > novel  (face>novel) > (shape>novel)  (shape>novel) > (face>novel) | 6 | 54 | MNI | yes | Table 3 |
| Dichter et al. (2012) | incentive delay | monetary incentives,  object image incentives | non-social | monetary anticipation (potential win vs non-win)  monetary outcome (win vs non-win)  object anticipation (potential win vs non-win)  object outcome (win vs non-win) | 24 | 53 | MNI | NA | Table 2, 3 |
| Dichter et al. (2012) | incentive delay | money rewards,  faces rewards | social | monetary anticipation  monetary outcome  face anticipation  face outcome | 17 | 51 | MNI | yes | Table 2, 3 |
| Duerden et al. (2013) | go/no-go | happy and sad faces | social | nogo – go | 2 | 2 | MNI | yes | Table 4 |
| Ewbank et al. (2017) | repetition suppression | faces | social | different identity > same identity | 1 | 0 | MNI | no | Page 97 |
| Fan et al. (2012) | attention network test - revised | arrows, asterisks | non-social | alerting (double cue vs baseline)  moving+engaging (valid cue - double cue)  orienting (valid+invalid spatial cue - double cue)  validity  disengaging  flanker conflict  alerting by flanker conflict  orienting by flanker conflict  validity by flanker conflict | 48 | 0 | MNI | no | Table 2 |
| Fan et al. (2014) | view body parts | images depicting hands and feet being injured or not | social | (solo pain + dyad pain) – (solo no-pain + dyad no-pain)  (dyad pain + dyad no-pain) – (solo pain + solo no-pain)  (dyad pain - dyad no-pain) – (solo pain - solo no-pain) | 17 | 0 | MNI | yes | Table s3-s5 |
| Gadgil et al. (2013) | hierarchical shape recognition | abstract shapes,  a simple gray square (control) | non-social | global > control  local > control  global > local | 0 | 5 | MNI | yes | Table 3 |
| Gaffrey et al. (2007) | semantic decision,  perceptual (control) | visual words,  unpronounceable consonants string (control) | non-social | semantic decision – perceptual control | 0 | 11 | Talairach | yes | Table 4 |
| Gebauer et al. (2014) | emotion rating | music | social | happy > sad | 0 | 3 | MNI | yes | Table 3 |
| Georgescu et al. (2013) | likeability rating | dynamically animated faces of virtual characters | social | direct gaze > averted gaze | 8 | 0 | MNI | yes | Table 2 |
| Gilbert et al. (2008) | random generation, alphabet | small square as a timing signal,  alphabet letters | non-social | baseline > random  stimulus-oriented > stimulus-independent | 4 | 17 | MNI | no | Page 2285, Table 5 |
| Grezes et al. (2009) | action perception | videos of static fearful, static neutral, dynamic fearful, dynamic neutral actions | social | (dynamic fearful + dynamic neutral) – (static fearful + static neutral)  (static fearful + dynamic fearful) – (static neutral + dynamic neutral)  (dynamic fearful - static fearful) – (dynamic neutral - static neutral) | 22 | 4 | MNI | no | Table 2-4 |
| Gu et al. (2015) | empathy for pain | painful and nonpainful scenarios | social | empathetic pain,  all images | 18 | 29 | MNI | yes | Table 3, s1 |
| Hadjikhani et al. (2014) | pain perception | short videos of pain and no-pain facial expressions | social | pain > no pain | 3 | 8 | MNI | no | Table 3 |
| Haist et al. (2005) | spatial attention | target letter, valid and invalid cues | non-social | short ISI (valid cue > baseline)  long ISI (valid cue > baseline) | 9 | 0 | Talairach | yes | Table 2, 3 |
| Hames et al. (2016) | sensory (audiovisual) | visual (three dots), auditory (three pure tones) | non-social | VV2 – VA2  AA2 – VV2  AA_MM2 – AA2 | 2 | 2 | MNI | yes | Figure 8-10 |
| Herrington et al. (2007) | biological motion perception | dots depicting a walking figure | non-social | walker – fixation  randomized walker – fixation | 45 | 0 | Talairach | yes | Table 3-4 |
| Hesling et al. (2010) | prosodic processing | audio prosodic connected speech (including intonation, rhythm, focus, and affect) | social | prosodic speech > rest | 0 | 1 | MNI | yes | Table 5 |
| Hsu et al. (2018) | simple conditioning | mimicking and anti-mimicking faces | social | mimicking > anti-mimicking | 1 | 0 | MNI | yes | Table 2 |
| Ishitobi et al. (2011) | emotional judgment,  figure discrimination (control) | positive and negative facial expressions (whole face),  scale images formed by squares or circles (control) | social | whole face > control | 2 | 0 | MNI | yes | Table 3 |
| Kana et al. (2006) | sentence comprehension | visual sentences with high- or low-imagery content | non-social | high imagery – fixation  low imagery – fixation | 14 | 12 | MNI | no | Table 1, 2 |
| Kana et al. (2007) | simple response inhibition,  response inhibition involving working memory | alphabets | non-social | simple inhibition – fixation  1-back inhibition – fixation | 16 | 2 | MNI | no | Table 2, 3 |
| Kana & Wadsworth (2012) | pun sentence comprehension | visual sentences | social | pun > fixation | 0 | 4 | MNI | yes | Table 2 |
| Kana et al. (2014) | physical and intentional causal attribution | comic strip vignettes depicting scenarios | social | intentional > physical | 4 | 0 | MNI | no | Table 2 |
| Kana et al. (2017) | semantic self-other processing | positive and negative visual trait adjectives | social | self – other  self – letter | 5 | 0 | MNI | no | Table 3 |
| Kennedy et al. (2006) | counting Stroop | emotional, neutral, and number words | non-social | number > fixation  emotional > neutral | 2 | 6 | Talairach | yes | Table 2 |
| Kennedy et al. (2008) | self- and other-reflection | statements referring to psychological personality traits (internal) or to observable external characteristics and behaviors (external) | social | external > internal | 0 | 4 | Talairach | yes | Table 4 |
| Kestemont et al. (2016) | causal attribution | sentences of positive and negative valences | social | positive self > truth baseline  positive other > truth baseline  positive situation > truth baseline  positive (self, other person, situation) > truth baseline  negative (self, other person, situation) > truth baseline | 0 | 7 | MNI | yes | Table 3 |
| Kleinhans et al. (2008) | letter fluency,  category fluency | letter (B, H, R, F),  category (animals, clothes, buildings, vehicles) | non-social | letter fluency – "nothing"  category fluency – "nothing" | 1 | 3 | Talairach | yes | Table 1 |
| Kleinhans et al. (2011) | supraliminal fearful face processing | pictures of fearful faces, houses, and scrambled mask | social | fear > scramble  house > scramble | 5 | 0 | MNI | yes | Table s1 |
| Kleinhans et al. (2016) | habituation | pictures of fearful faces, houses, and scrambled mask | social | house 1 > house 2 | 2 | 0 | MNI | yes | Table s3 |
| Koshino et al. (2008) | n-back working memory (involving face recognition) | faces | social | working memory > fixation | 20 | 3 | MNI | no | Table 4 |
| Lassalle et al. (2017) | view faces | low and high intensity pictures of fearful, angry, happy, and neutral faces | social | fearful40 > neutral  happy100 > neutral  angry100 vs angry40  fearful100 vs fearful40  happy100 vs happy40 | 70 | 42 | MNI | yes | Table 2-3, s10-12 |
| Lassalle et al. (2019) | view video (pain/disgust perception) | video clips depicting body parts in painful, disgusting, and neutral condition | social | painful > neutral  disgusting > neutral | 13 | 0 | MNI | yes | Table 4, 5 |
| Marsh et al. (2011) | watch hand-action movies | goal-directed actions (rational action, rational action with barrier, irrational action, irrational action with barrier),  moving shapes (control) | non-social | hand actions > shapes  simple, straight hand actions > shapes | 15 | 0 | MNI | yes | Table s2, s5 |
| Martineau et al. (2010) | hand motion perception | videotape of a hand performing a flexion-extension movement,  static hand picture (control rest) | non-social | observation > rest | 0 | 2 | MNI | yes | Table 1 |
| Mizuno et al. (2011) | perspective taking (requiring deictic shifting) | opened book and closed book scenes | social | all task conditions – fixation | 0 | 6 | MNI | no | Table 3 |
| Morita et al. (2012) | self-face processing | images or self and others' faces | social | self > others | 3 | 0 | MNI | yes | Table 3 |
| Morita et al. (2016) | self-face processing | images or self and others' faces | social | self > others | 2 | 0 | MNI | yes | Table 2 |
| Moseley et al. (2015) | silent reading | abstract emotion words, animal names, abstract verbs | non-social | emotion words  abstract verbs  animal names | 46 | 0 | MNI | yes ^†^ | Table 1, s3 |
| Muller et al. (2001) | visually paced finger movement,  view movement (control) | a diagram of a hand with a blue dot appearing | non-social | task block – control block | 13 | 13 | Talairach | yes | Table 3 |
| Muller et al. (2003) | visuomotor learning | a diagram of a hand with a blue dot appearing | non-social | random – same,  random – sequence | 26 | 18 | Talairach | yes | Table 3 |
| Muller et al. (2004) | visuomotor learning | a diagram of a hand with a blue dot appearing | non-social | early learning  late learning | 24 | 23 | Talairach | yes | Table 4 |
| Murphy et al. (2014) | sustained attention | visual stimuli (timer displaying RTs after predictable or unpredictable delays) | non-social | all delays > baseline | 11 | 0 | Talairach | NA | Table 2 |
| Murphy et al. (2017) | temporal discounting | monetary reward (small, variable, immediate or large, fixed, delayed) | non-social | delayed reward > immediate reward | 4 | 0 | Talairach | NA | Table 2 |
| Nijhof et al. (2018) | mentalizing | short video animations | social | false belief > true belief | 1 | 0 | MNI | yes | Table 2 |
| Ohta et al. (2012) | visual target detection | rapid serial visual presentation of T-shaped stimuli | non-social | (LD+ and HD+) > (LD- and HD-)  (LD+ vs LD-) > (HD+ vs HD-) | 1 | 3 | MNI | no | Table 3, Figure 5 |
| Okamoto et al. (2018) | hand observation | self or others' hands in first- or third-person perspective | social | third > first | 2 | 0 | MNI | yes | Table 2 |
| Pantelis et al. (2015) | watch TV show with social awkwardness | an episode of the TV show 'The Office' | social | awkward > baseline | 3 | 0 | MNI | NA | Page 1352 |
| Perkins et al. (2015) | action observation | video tasks (hand-object manipulations, hand-mouth interactions, hand-communicative actions, hand-directive actions) | non-social | action observation > baseline | 3 | 2 | MNI | yes | Table 1, Figure 2 |
| Perlman et al. (2011) | free viewing of faces | a fearful face | social | free viewing | 3 | 0 | Talairach | yes | Figure 2 |
| Poulin-Lord et al. (2014) | visuo-motor imitation | hand gestures | social | both conditions (left and right hand)  left hand condition  right hand condition | 11 | 17 | MNI | yes | Table 4 |
| Redcay et al. (2013) | joint attention | Catch the Mouse' game | social | responding to joint attention > solo attention | 2 | 3 | MNI | yes | Table 3 |
| Richey et al. (2015) | cognitive reappraisal | faces (neutral closed-mouth images) | social | enhance positive > pre-instruction  enhance negative > pre-instruction  any regulation > pre-instruction | 7 | 0 | MNI | yes | Table 2 |
| Rosenblau et al. (2017) | emotional prosody processing | semantically neutral sentences spoken with emotional or neutral prosody | social | complex > basic emotional prosody  implicit > explicit emotional prosody | 33 | 0 | MNI | yes | Table 5 |
| Sabatino et al. (2013) | visual target detection | faces,  non-social high autism interest images | non-social | face  high autism interest  high autism interest vs face | 17 | 3 | MNI | yes ^†^ | Table 2, 3 |
| Samson et al. (2015) | controlled listening | pure and harmonic tones | non-social | all sound – silence baseline | 20 | 31 | MNI | yes | Table s2 |
| Sato et al. (2017) | gaze-triggered attentional orienting | averted or straight eye gaze cue followed by a target | social | supraliminal (averted > straight eyes)  subliminal (averted > straight eyes) | 4 | 0 | MNI | yes | Table 3 |
| Schelinski et al. (2016) | voice identity recognition,  speech content recognition | a spoken sentence followed by a stream of 12 two-word spoken sentences | social | vocal sounds > silence baseline  non-vocal sounds > vocal sounds  voice identity > silence baseline  voice identity > speech  speech > silence baseline | 19 | 1 | MNI | yes ^†^ | Table s3 |
| Schipul et al. (2016) | implicit prototype learning | white dots on a black background | non-social | encoding – fixation  early encoding – late encoding | 19 | 20 | MNI | no | Table s1, s2 |
| Schmitz et al. (2006) | go/no-go,  stroop,  switch | arrows,  four dots,  a double-headed arrow (horizontal or vertical) | non-social | no-go – oddball  incongruent – congruent  switch – repeat | 0 | 5 | Talairach | yes | Table 5 |
| Schmitz et al. (2008) | continuous performance with monetary incentive | letter stream | non-social | rewarded – non-rewarded | 0 | 2 | Talairach | yes ^†^ | Figure 1 |
| Schneider et al. (2013) | moral reasoning | short textual dilemma situations | social | moral > BL  moral txt > BL txt  SE > BL  IND > BL  IND > SE | 5 | 11 | MNI | yes | Table 4, s4 |
| Schulte-Ruther et al. (2011) | empathic social cognition | emotional facial expressions (happy, sad),  neutral faces (control) | social | other – control  self – control | 2 | 4 | MNI | yes | Table 3 |
| Shafritz et al. (2008) | target detection (shifts in response/cognitive set) | geometric shapes (squares, triangles, or circles) | non-social | all target trials  target-shift trials  target-maintain trials  novel trials | 16 | 0 | Talairach | no | Table 1, Page 6 |
| Shafritz et al. (2015) | go/no-go | letters; happy, fearful, and neutral faces | social | letter nogo – letter go  happy go – fear go  fear nogo – happy nogo  happy nogo – fear nogo  emotional nogo – letter nogo | 3 | 7 | MNI | yes | Table 4 |
| Silani et al. (2008) | emotional rating (internal),  physical judgment (external) | full-color pictures (unpleasant, neutral, and pleasant) | social | internal > external  unpleasant > neutral  internal (unpleasant-neutral) > external (unpleasant-neutral) | 15 | 10 | MNI | yes ^†^ | Table s1-s3 |
| Simard et al. (2015) | Raven's Standard Progressive Matrices (fluid reasoning) | matrices each with a missing entry and 8 choices | non-social | figural < analytical < complex analytical | 3 | 2 | MNI | no | Table 3 |
| Solomon et al. (2015) | reinforcement learning (with probabilistic feedback) | three pairs of Japanese characters | non-social | feedback  early stimulus  stimulus | 4 | 3 | Talairach | yes | Page 7 |
| Sommer et al. (2018) | object transfer false belief | animation | social | question phase | 2 | 5 | MNI | yes | Table 3 |
| Soulieres et al. (2009) | Raven's Standard Progressive Matrices (matrix reasoning),  pattern matching (control) | matrices of geometric designs with eight options | non-social | RSPM > fixation  pattern matching > fixation | 5 | 13 | MNI | no | Table 4, 6 |
| Stanfield et al. (2017) | social judgment,  gender judgment | faces | social | social > gender  gender > baseline | 2 | 2 | MNI | no | Table s7, Page 1225 |
| Takarae et al. (2007) | visually guided saccade,  smooth pursuit | moving circle | non-social | visually guided saccade  smooth pursuit | 30 | 19 | Talairach | NA | Table 2 |
| Tanabe et al. (2012) | real-time joint attention | balls | social | (ES' + EN') – (BS' + BN') | 1 | 0 | MNI | yes | Table 2 |
| Tietze et al. (2019) | audiovisual speech perception | congruent and incongruent audiovisual (lip movement matched or did not match spoken word) | non-social | NA | 1 | 0 | Talairach | yes | Page 4 |
| Travers et al. (2015) | motor sequence learning | four circles and an airplane drawing | non-social | sequenced > nonsequenced | 1 | 0 | MNI | yes | Figure 3 |
| Velasquez et al. (2017) | go/no-go | letters and faces (happy and sad) | social | letter nogo > go  face nogo > go  face nogo > letter nogo | 3 | 6 | MNI | yes | Table 3 |
| Wicker et al. (2008) | emotional judgment,  age judgment | angry and happy faces of young or older actors, with direct or averted gaze | social | emotion vs age | 3 | 0 | Talairach | yes | Table 3 |
| Yamada et al. (2012) | Raven's Standard Progressive Matrices (geometric reasoning) | matrices with six options | non-social | easy analytic – baseline | 0 | 1 | MNI | yes | Figure 3 |
| Zurcher et al. (2013) | grotesqueness perception | thatcherized eyes or mouth, upright and inverted faces | social | eyes upright > eyes inverted  eyes inverted > eyes upright | 24 | 3 | MNI | yes | Table 3 |
| Zurcher et al. (2013) | social observation | fearful faces | social | averted gaze > direct gaze | 37 | 0 | MNI | yes | Table 3 |

NA = not specified, ^†^ = uncorrected comparisons also included in the meta-analysi
